# Regional and Country Prevalence Estimates of Unsafe Sex Among Adolescents in 68 Low-Income and Middle-Income Countries

**DOI:** 10.1007/s10508-024-02861-1

**Published:** 2024-04-18

**Authors:** Janni Leung, Carmen Lim, Habte Belete, Caitlin Mcclure-Thomas, Shaun Foo, Gary Chung Kai Chan

**Affiliations:** 1 Faculty of Health and Behavioural Sciences, National Centre for Youth Substance Use Research, The University of Queensland, 31 Upland Road, St. Lucia, QLD 4067 Australia; 2 Department of Psychiatry, Bahir Dar University, Bahir Dar, Ethiopia

**Keywords:** Adolescents, Unsafe sex, Prevalence, Global health, Low-income countries, Sexual intercourse

## Abstract

Prioritizing adolescent health is a public health priority to achieve the sustainable development goals, including reducing the risk of unsafe sex. Data on unsafe sex have remained scarce among adolescents in low-and middle-income countries (LMICs). To estimate the prevalence of unsafe sex in LMICs, we conducted secondary data analysis on the Global School-based Student Health Surveys among 244,863 students aged 13–17 years from 68 countries across five World Health Organization regions. The overall prevalence of ever had sex was 16.2%. The highest to lowest regional prevalence estimation of ever had sex was 30.5% (28.9–32.1) in the Americas, 28.6% (26.8–30.4) in Africa, 10.9% (9.2–12.6) in the Eastern Mediterranean, 9.6% (8.8–10.5) in South-East Asia, and 8.0% (6.8–9.1) in the Western Pacific. The highest prevalence of sexual intercourse before age 14 and practicing sexual intercourse without condom use were 36.5% (34.5–38.5) and 32.2% (30.1–34.3) in Africa, respectively. Findings suggest that current interventions are inadequate in promoting the uptake of safe sexual behaviors and an urgent intervention is needed.

**Supplementary Information:**

The online version contains supplementary material available at 10.1007/s10508-024-02861-1.

## Introduction

Unsafe sex is a global health concern, and wherein one million deaths annually can be attributed to unsafe sex (Murray et al., 2020). Unsafe sex refers to high-risk sexual behaviors, including practicing sex at an early age, having multiple sexual partners, unprotected sex through no condom use and no birth control/contraceptives (Alimoradi et al., 2017; Nkata et al., 2019; Shayo & Kalomo, 2019). Unsafe sex is the fifth leading behavioral risk factor for all causes of disease burden in 2019, with over 46 million disability-adjusted life years attributable to it (Murray et al., 2020).

Data on unsafe sex remain limited in low- and middle-income countries (LMICs) where vulnerable populations (e.g., adolescents) may be disproportionately affected (Global Burden of Disease (GBD), 2019). Adolescence is the life period in which individuals are at increased vulnerability to external and peer influences and begin to engage in risk-taking behaviors (Madise et al., 2007). To protect the health of adolescents, the United Nations has made it a public health priority to eradicate disparities in sexual and reproductive health by 2030, as stipulated by the sustainable development goal (SDG 3)–where adolescents must have the right to comprehensive sexuality education, and universal access to contraceptive information and services (World Health Organization, 2023a).

Substantial variations in prevalence of unsafe sex are observed between high- and low-income countries. For example, in the USA, 3.9% reported having sex before age 13 (Centers for Disease Control and Prevention, 2022). In comparison, in Mozambique, 41.5% reported having early sex initiation (Pengpid & Peltzer, 2021). Such disparities may be attributed to factors that increase the risk of unsafe sexual behaviors in LMICs, such as cultural factors in low-income countries or social influences such as influenced by peer pressure, sexual experimentation, myths about contraception, poor parental supervision, poverty leading to unsafe sex, fear of partner rejection, and access barriers to reproductive health education (Govender et al., 2020).

Understanding the epidemiology of unsafe sex in LMICs is a global concern because more than half of the unintended pregnancies (World Health Organization, 2022b) and HIV human immunodeficiency virus (HIV) prevalence in adolescents are from low-middle-income countries. Unsafe sex is also associated with a range of negative health outcomes, including increased risk of transmitting HIV and other sexually transmitted infections (STI) (Kazdouh et al., 2019), unintended pregnancies, abortions (Mahumud et al., 2022), and psychological issues including suicidality (Chernick et al., 2020).

Limited data exist on unsafe sex prevalence in Low- and Middle-Income Countries (LMICs). In 43 LMICs, 52.0% of adolescents (12–15 years) had two or more sexual partners, and 44.2% did not use condoms (Smith et al., 2022). Similarly, in four Caribbean countries (2016–2017), 58.8% had multiple sexual partners, 58.6% engaged in early sexual intercourse, 41.9% lacked birth control, and 28.4% did not use condoms (Pengpid & Peltzer, 2020). Despite these findings, a comprehensive understanding of unsafe sex, incorporating more countries, diverse survey years, and a broader age range, remains underexplored. Given this, there is a need to elucidate the country-specific prevalence of unsafe sexual behaviors among adolescents residing in LMICs.

Previous studies have largely been restricted to a single or a few countries. For example, in a study of four Southeast Asian countries (Pengpid & Peltzer, 2020), the prevalence of having multiple sex partners was found to vary widely between countries, ranging from 16.1% in Laos to 73.9% in Indonesia. This further highlights the need to assess a larger pool of country-specific estimates using the same sampling approach and survey instrument such as the Global School-based Student Health Survey (GSHS).

Therefore, the current study aimed to assess the prevalence of unsafe sexual behaviors in adolescents aged 13–17 years in 68 countries across five World Health Organization (WHO) regions using the GSHS data (World Health Organization, 2023b).

## Method

### Participants

We used data from the GSHS implemented in 68 countries. GSHS are national school-based cross-sectional surveys among students aged 13–17 years (Centers for Disease Control and Prevention, 2018).The purpose of the GSHS surveys is to gather data on adolescent health behaviors and protective factors to support school and youth programs and policies globally. Questionnaires contain modules on alcohol, dietary behavior, drug use, mental health, hygiene, physical activity, protective factors, and tobacco use. All surveys used the same selection process, survey methodology, and standardized questionnaire (Centers for Disease Control Prevention, 2013). We included 244 863 adolescents from 68 countries. Sample sizes range from 140 in Tokelau to 28,368 in Argentina.

### Procedure

Data used in the paper come from GSHS which is a complex survey which employs a two-stage, cluster sampling design to select representative sample of middle-school students in each country. In the first stage of sampling, schools are chosen based on probability proportional to enrolment size. In the second stage, specific classes are randomly selected. All students in these classes are eligible to participate. As the aim of the current analysis focuses on groups of young adolescents aged 13–17 years, this report aligns the survey procedure including the publicly available question responses, the cross-country comparability by considering the national representativeness of each survey, as well as the probability of school and classroom selection. Finally, a self-administered questionnaire was given to all students in the selected classroom. Parental consent and student assent are obtained. The ethics application was approved by the WHO. Additional details on the survey can be found here (Centers for Disease Control Prevention, 2013): https://www.cdc.gov/gshs/index.htm

Participants were from the five WHO regions: Africa region, Americas, Eastern Mediterranean Region, Western Pacific region, and South-East Asia (World Health Organization, 2023b). A total of 73 countries and territories administered the sexual risk module but only data from 68 countries could be used.

Of the total 68 countries, 16 (23.53%) were from Africa region, 28 (41.18%) Americas, 3 (4.41%) Eastern Mediterranean region, 15 (22.06%) Western Pacific region, and 6 (8.82%) South-East Asia.

Surveys lacking the outcome variable and exhibiting inconsistencies were excluded. As a result, the following surveys were excluded from our analysis because no data were available or not national representative (Colombia, Ecuador, United Arab Emirates, Venezuela, Zimbabwe). Response rates for each country range between 57% in Vanuatu to 97% in Swaziland (Eswatini).

### Measures

The outcome variable, unsafe sex, was assessed through respondents’ self-disclosed engagement in risky sexual practices. These practices encompassed a lifetime history of sexual activity, initiation of sexual activity before reaching 14 years of age, involvement with multiple sexual partners, engaging in sexual activity without the use of condoms, and absence of birth control measures.

In each survey, the five binary outcome variables were coded as follows: (1) ever had sexual intercourse (coded 1 = yes, 0 = no), (2) early sexual initiation before age 14 (coded 1 = 14 years or younger, 0 = 15 + years), (3) having multiple sex partners (coded 0 = 1 or never had sex, 1 = 2 or more), (4) no condom use (coded 1 = no condom use, 0 = yes or never had sexual intercourse), and (5) no birth control (coded 1 = no birth control, 0 = yes or never had sexual intercourse).

### Statistical Analysis

Prevalence of the unsafe sex variables was estimated overall, in each region, and in each country. We used the SURVEY procedure in SAS version 9.4 to account for complex sampling design in GSHS study (e.g., survey clustering, strata and primary sampling unit). The prevalence estimates were multiplied by the population sizes of the countries (age 13–17) to calculate the number of adolescents affected. Bubble plots were generated to visualize the unsafe sex variables against the prevalence of adolescents who had ever had size, with the size of the dots proportional to the number of cases of adolescents with each of the unsafe sex risk factors.

## Results

### Prevalence of Adolescents Who Ever Had Sex

Overall, out of 244,863 participants, 58,666 (16.2%) participants reported ever having sexual intercourse (Table 1). The prevalence of ever-had sex varied by region (Table 1). In Africa, Mozambique reported the highest ever having sexual intercourse rate at 50.7% (43.5–57.9), while the United Republic of Tanzania had the lowest at 19.0% (16.7–21.2). In the Eastern Mediterranean Region, Djibouti reported the highest ever having sexual intercourse rate at 34.7% (31.7–37.8), while Tajikistan had the lowest at 8.7% (6.9–10.6).

**Table 1:** Sample characteristics and prevalence of students who had ever had sex in the Global School-based Student Health Surveys (*n* = 244,863)

Country	Survey year	Response rates	Sample size	Ever had sexual intercourse % (95% CI)		
				All	Age 12–15	Age 16+
*Africa*						
Benin	2016	78	2536	49.9 (45.7–54.1)	23.5 (18.3–28.7)	58.6 (54.7–62.5)
Botswana	2005	95	2197	19.2 (16.9–21.6)	14.7 (12.5–17)	27.4 (23–31.9)
Ghana	2012	77	3632	31.7 (27.2–36.3)	24.6 (18.9–30.3)	37.7 (32.8–42.6)
Kenya	2003	84	3691	27.0 (24.4–29.7)	24.6 (22.3–26.9)	39.2 (34.1–44.4)
Malawi	2009	94	2359	22.9 (18.2–27.6)	22.8 (18.1–27.5)	49.6 (20.8–78.4)
Mauritania	2010	70	2063	29.7 (25.1–34.3)	26.9 (21.3–32.6)	33.8 (28.8–38.7)
Mauritius	2017	84	3012	19.7 (16.3–23)	14.9 (12.4–17.5)	27.5 (21.4–33.7)
Namibia	2013	89	4531	47.1 (43.8–50.5)	31.9 (28.7–35.2)	57.8 (54.3–61.4)
Senegal	2005	60	3154	22.0 (17.3–26.6)	19.2 (15.3–23)	43 (37.7–48.3)
Seychelles	2015	82	2540	37.9 (34.4–41.4)	34 (30.4–37.7)	55.2 (48.6–61.8)
Swaziland	2013	97	3680	26.0 (23.6–28.4)	13.4 (11.5–15.4)	33.6 (30.4–36.8)
Uganda	2003	69	3215	20.4 (17.5–23.2)	15.4 (12.9–18.0)	28.4 (24.4–32.3)
United Republic of Tanzania	2014	87	3793	19.0 (16.7–21.2)	17.7 (15.1–20.2)	24.2 (20.8–27.5)
Zambia	2004	70	2257	22.4 (19.9–24.9)	19.9 (16.7–23.2)	26.6 (22.5–30.6)
Liberia	2017	71	2744	49.8 (46.4–53.2)	30.7 (26–35.3)	56.1 (52.6–59.5)
Mozambique	2015	80	1918	50.7 (43.5–57.9)	38.2 (30.3–46)	63.9 (58.6–69.1)
Africa total	–	–	47,322	28.6 (26.8–30.4)	21.8 (20.2–23.4)	42 (39.4–44.6)
*Region of Americas*						
Anguilla	2016	88	813	32.2 (26.9–37.5)	27.6 (21.8–33.3)	44.2 (36.5–51.9)
Antigua and Barbuda	2009	67	1266	33.9 (29.4–38.5)	33.9 (29.3–38.4)	37.6 (12.4–62.9)
Argentina	2012	71	28,368	40.0 (37.2–42.9)	34.6 (31.4–37.8)	62.3 (59.4–65.2)
Barbados	2011	73	1629	33.3 (29.6–37)	32 (28.2–35.7)	49.2 (37–61.4)
Belize	2011	88	2112	23.5 (19.9–27.2)	19.1 (16.2–21.9)	44.1 (36.6–51.6)
Bolivia	2012	88	3696	24.9 (21.5–28.2)	19.6 (17.6–21.7)	41.4 (35.2–47.6)
British Virgin Islands	2009	90	1664	37.8 (37.8–37.8)	30.5 (30.5–30.5)	58.6 (58.6–58.6)
Cayman Islands	2007	79	1299	37 (37–37)	35.9 (35.9–35.9)	44.7 (44.7–44.7)
Chile	2013	60	2049	37.9 (32.6–43.2)	23.5 (20.1–26.9)	56.8 (48.7–65)
Costa Rica	2009	72	2679	23.5 (19.3–27.7)	18 (15.8–20.1)	51.4 (42.1–60.7)
Dominica	2009	84	1642	45 (40.3–49.6)	39.4 (35.3–43.5)	67.4 (60.8–74)
Grenada	2008	78	1542	42.6 (37.6–47.6)	36 (32.9–39)	73.3 (65–81.5)
Guatemala	2015	82	4374	19.5 (16.2–22.8)	15.4 (12.9–17.9)	38.6 (29.3–47.9)
Guyana	2010	76	2392	31.8 (27–36.7)	29.3 (25.1–33.5)	45.8 (36.4–55.3)
Honduras	2012	79	1779	24.7 (22.2–27.3)	21.4 (18.8–24)	45.6 (40–51.3)
Jamaica	2017	60	1667	46.8 (40.6–53)	36.9 (30.8–43)	58.1 (51.3–64.9)
Peru	2010	85	2882	19.4 (16.7–22)	16.8 (14.6–19)	32.1 (25.5–38.6)
Saint Kitts and Nevis	2011	70	1740	36.1 (36.1–36.1)	30.8 (30.8–30.8)	59.6 (59.6–59.6)
Saint Lucia	2007	77	1276	34.2 (29.8–38.5)	30.6 (26.2–34.9)	53.5 (45–62.1)
Saint Vincent and the Grenadines	2007	84	1333	39.9 (33.7–46.1)	37.8 (31.2–44.4)	61.7 (52.8–70.6)
Suriname	2016	83	2126	32.3 (23.4–41.2)	19 (14.1–23.9)	60.6 (52–69.1)
Trinidad and Tobago	2017	89	3869	26.2 (22.4–30)	21.7 (18.5–25)	39.1 (31–47.3)
Uruguay	2012	77	3524	33.7 (30.6–36.9)	27.5 (24.9–30.1)	64.2 (58.9–69.6)
Bahamas	2013	78	1357	28.2 (24–32.4)	26.4 (22.8–30.1)	71.8 (47.3–96.3)
El Salvador	2013	88	1915	22.4 (19.2–25.7)	18.6 (16–21.1)	45.8 (36.7–54.9)
Curacao	2015	83	2765	36.9 (32.3–41.5)	18.7 (16–21.3)	55.7 (51.1–60.2)
Paraguay	2017	87	3149	28.6 (24.4–32.8)	16.8 (14.3–19.3)	47.4 (42.5–52.2)
Dominican Republic	2016	63	1481	40.7 (32.8–48.5)	32.4 (26.2–38.7)	48.3 (37.5–59.1)
Americas total	–	–	86,388	30.5 (28.9–32.1)	23.2 (22.1–24.3)	50.6 (47.4–53.8)
*Eastern Mediterranean region*						
Tajikistan	2006	80	9714	8.7 (6.9–10.6)	9.2 (7–11.3)	7.1 (5–9.2)
Macedonia	2007	–	2114	15.9 (11.8–19.9)	12.4 (9.4–15.4)	29 (21.3–36.6)
Djibouti	2007	83	1777	34.7 (31.7–37.8)	29.7 (25.6–33.8)	39.4 (36.2–42.7)
Eastern Mediterranean total	–	–	13,605	10.9 (9.2–12.6)	10.2 (8.5–12)	12.8 (10.1–15.5)
*South-East Asian region*						
Indonesia	2015	94	11,142	5.8 (4.7–6.9)	6 (4.7–7.2)	4.6 (3.4–5.9)
Thailand	2015	89	5894	19.1 (17.2–20.9)	14.8 (12.3–17.4)	28.4 (23.5–33.3)
Bangladesh	2014	91	2989	8.2 (6–10.3)	8.6 (6.4–10.9)	3.1 (0.2–6)
Bhutan	2015	79	7576	17.1 (15.5–18.7)	11.7 (10.4–13.1)	21.5 (19.5–23.5)
Timor Leste	2016	95	3704	22.1 (19.5–24.8)	18.1 (14.4–21.8)	24.8 (21.8–27.8)
Nepal	2015	69	6529	19.2 (16–22.5)	18 (14.6–21.4)	22.1 (18.2–26.1)
South-East Asia total	–	–	37,834	9.6 (8.8–10.5)	8.8 (7.8–9.8)	12.9 (10.1–15.6)
*Western Pacific Region*						
Cambodia	2013	85	3806	11.6 (10.1–13.2)	12.6 (10.5–14.7)	10.6 (8.7–12.5)
Fiji	2016	79	3705	21.3 (16.1–26.6)	16.8 (12–21.5)	25.1 (18.5–31.7)
Kiribati	2011	85	1582	23.5 (20.7–26.4)	21.2 (18.8–23.7)	36.4 (28.8–44)
Malaysia	2012	89	25,507	8.2 (7.4–8.9)	7.9 (7–8.9)	8.5 (7.5–9.5)
Mongolia	2013	88	5393	15.1 (12.8–17.5)	9.6 (8.2–10.9)	26.3 (22–30.7)
Nauru	2011	73	578	31.7 (31.7–31.7)	28.1 (28.1–28.1)	43.4 (43.4–43.4)
Samoa	2017	59	1955	25 (21.4–28.7)	21.3 (17.4–25.2)	29.7 (25–34.3)
Vanuatu	2016	57	2159	26.8 (23.5–30.2)	21.4 (18.3–24.5)	33.8 (28–39.5)
Vietnam	2013	96	3331	6.7 (5–8.4)	3.9 (3–4.9)	8.8 (6.1–11.6)
Brunei	2014	65	2599	11.4 (9.9–12.8)	10 (8.3–11.6)	14.8 (11.6–18)
Tuvalu	2013	90	943	18.6 (18.6–18.6)	16.1 (16.1–16.1)	24.1 (24.1–24.1)
French Polynesia	2015	70	3216	40.1 (34.1–46.1)	28.4 (24.8–32)	61.8 (56.9–66.6)
Laos	2015	70	3683	14.5 (12.2–16.8)	9.2 (7.3–11.1)	17.9 (15–20.8)
Tokelau	2014	71	140	30.2 (21.9–38.5)	19.4 (13.4–25.4)	63.1 (49.9–76.2)
Wallis and Futuna	2015	82	1117	26.8 (23.3–30.3)	20.8 (16.8–24.8)	36.8 (32–41.6)
Western Pacific total	–	–	59,714	8.0 (6.8–9.1)	6.1 (5.3–7)	9.7 (7.7–11.7)
Total	–	–	244,863	16.2 (15.4–17)	13.1 (12.3–13.9)	23.2 (21.1–25.3)

In South-East Asia, Timor Leste reported the highest ever having sexual intercourse rate at 22.1% (19.5–24.8), while Indonesia had the lowest at 5.8% (4.7–6.9). In the Western Pacific region, French Polynesia reported the highest ever having sexual intercourse rate at 40.1% (34.1–46.1), while Vietnam had the lowest at 6.7% (5.8–4.0).

Among adolescents aged 16 and older, the Americas had the highest prevalence at 50.6% (47.4–53.8), followed by Africa at 42.0% (39.4–44.6). The Western Pacific region had the lowest prevalence at 9.7% (7.7–11.7), followed by the Eastern Mediterranean Region at 12.8% (10.1–15.5), and South-East Asia at 12.9% (10.1–15.6).

For adolescents aged 12–15, the Americas had the highest prevalence at 23.2% (22.1–24.3), followed by Africa at 21.8% (20.2–23.4). The Western Pacific region had the lowest prevalence at 6.1% (5.3–7.0), followed by South-East Asia at 8.8% (7.8–9.8), and the Eastern Mediterranean Region at 10.2% (8.5–12.0) (Table 1).

### Prevalence of Unsafe Sex

The overall prevalence of sexual intercourse before age 14 was 31.4% (30–32.9). The prevalence of having multiple sex partners was 33.2% (31.8–34.6). Prevalence of no condom use was 27.4% (25.9–28.8) and 21.2% (20.1–22.4) was no birth control used (Table 2).

**Table 2:** Regional- and country-level prevalence of unsafe sex and high-risk sexual behaviors

	Prevalence of unsafe and high-risk sex among those who ever had sex % (95%CI)			
	Sexual intercourse before age 14	Multiple sex partners	No condom use during sexual intercourse	No birth control methods during sexual intercourse
Total	31.4 (30–32.9)	33.2 (31.8–34.6)	27.4 (25.9–28.8)	21.2 (20.1–22.4)
*Regional-level estimates*				
Africa	36.5 (34.5–38.5)	34.5 (32.5–36.5)	32.2 (30.1–34.3)	19.2 (17.5–20.9)
Region of Americas	35.2 (33–37.3)	46.7 (44.6–48.8)	28.3 (26.5–30)	32.7 (31–34.4)
Eastern Mediterranean Region	36.3 (31.2–41.4)	30.6 (26.6–34.6)	18.6 (15–22.2)	–
South-East Asian Region	26.8 (23.4–30.2)	31.7 (18.9–24.4)	20.9 (17.5–24.3)	15.3 (13.3–17.3)
Western Pacific Region	14.9 (11.9–17.9)	21.8 (18–25.7)	24.3 (19.3–29.3)	14.6 (11.2–17.9)
*Country-level estimates*				
Africa				
Benin	23.4 (18.9–28)	61.2 (57.3–65.1)	47.3 (41.8–52.8)	41.2 (37.9–44.5)
Botswana	43.7 (37.8–49.7)	45.6 (39.4–51.9)	27.5 (23.3–31.6)	–
Ghana	25.6 (21.9–29.4)	33.8 (30–37.5)	33.2 (28.1–38.3)	27.8 (23.6–32.1)
Kenya	68.9 (64–73.9)	-	49.2 (45.4–53.1)	–
Malawi	40.2 (33.2–47.2)	22.5 (14.2–30.9)	13.8 (9.2–18.4)	–
Mauritania	33.8 (28.7–38.8)	33.9 (27.4–40.5)	21.5 (16–27)	18.5 (13.5–23.5)
Mauritius	27 (22.1–31.8)	38.9 (32.7–45)	35.2 (29.1–41.4)	39 (34.9–43)
Namibia	32.9 (29.4–36.3)	49.3 (46–52.5)	19.5 (16.9–22.1)	30.7 (27.5–34)
Senegal	65.3 (55.9–74.8)	58.2 (52.6–63.8)	37.5 (27.3–47.7)	–
Seychelles	51.4 (47.1–55.8)	53.7 (49.5–57.9)	38.8 (34.8–42.8)	48.7 (44.1–53.2)
Swaziland	–	44.8 (41.4–48.3)	25 (22–28.1)	36.9 (33.9–39.9)
Uganda	46.3 (40.8–51.7)	48.1 (44.1–52.1)	32.4 (26.8–38)	–
United Republic of Tanzania	37.2 (31.7–42.6)	26.9 (23–30.8)	29.3 (24.5–34.1)	17.6 (14.1–21.1)
Zambia	53.2 (46.5–59.9)	47.4 (43.6–51.2)	34.6 (28.6–40.5)	–
Liberia	15.1 (12.3–18)	45.1 (41.6–48.6)	27.5 (24.8–30.3)	20.5 (17.4–23.6)
Mozambique	28.4 (24.7–32.2)	40.8 (35–46.7)	17.5 (14.2–20.8)	23.4 (18.7–28.1)
Region of Americas				
Anguilla	44.4 (35.9–52.9)	47.8 (41.3–54.2)	28.4 (23.3–33.5)	39.4 (33.8–44.9)
Antigua and Barbuda	67.3 (62–72.6)	62.6 (58.2–67)	27.7 (23.1–32.3)	51.3 (46.8–55.9)
Argentina	41.6 (39–44.2)	48.5 (45.9–51)	19.6 (17.4–21.8)	30.8 (27.9–33.8)
Barbados	51.3 (46.1–56.4)	48.3 (43.2–53.3)	29 (25.4–32.6)	45.8 (41.5–50)
Belize	45.3 (40.4–50.2)	52.7 (47.7–57.7)	24.3 (20–28.6)	37.1 (31.9–42.2)
Bolivia	30.5 (25.7–35.3)	39.4 (35.3–43.4)	29.4 (25.7–33.1)	29.2 (26–32.3)
British Virgin Islands	58 (58–58)	58.4 (58.4–58.4)	23.9 (23.9–23.9)	35.2 (35.2–35.2)
Cayman Islands	64.7 (64.7–64.7)	52.8 (52.8–52.8)	24 (24–24)	–
Chile	23.4 (17.6–29.1)	44.5 (36.9–52.1)	39 (33.6–44.4)	33.5 (27.9–39.1)
Costa Rica	36.8 (32.6–41)	44.7 (35.7–53.7)	32 (25.3–38.7)	25 (21.4–28.5)
Dominica	60.9 (56.1–65.7)	59 (54.7–63.2)	29.7 (25.9–33.4)	52.8 (48.1–57.5)
Grenada	61.7 (57.8–65.6)	55.2 (50.5–59.9)	30.4 (26.5–34.2)	–
Guatemala	31.5 (24.6–38.5)	41.9 (32.5–51.3)	29.6 (22.7–36.4)	31.8 (25.6–38.1)
Guyana	47.6 (43.4–51.8)	46.8 (44–49.6)	23.4 (20.6–26.3)	–
Honduras	39.5 (33.8–45.2)	34.7 (31–38.4)	23.5 (19.7–27.2)	26.8 (22.4–31.2)
Jamaica	53.7 (48.1–59.3)	63.6 (59.7–67.5)	30.4 (26–34.8)	44 (39–48.9)
Peru	41.8 (37.6–46.1)	46.9 (43.1–50.6)	32.6 (28.9–36.2)	41.3 (36.9–45.8)
Saint Kitts and Nevis	52.5 (52.5–52.5)	58.5 (58.5–58.5)	37.1 (37.1–37.1)	46.4 (46.4–46.4)
Saint Lucia	60 (52.8–67.1)	55.5 (50.7–60.3)	37.4 (31.9–42.8)	–
Saint Vincent and the Grenadines	68.8 (63.7–73.8)	55.2 (49.8–60.6)	30.8 (26.1–35.5)	–
Suriname	33.1 (26.5–39.8)	50.3 (43.8–56.7)	30 (24.7–35.2)	40.4 (36.9–43.8)
Trinidad and Tobago	37.3 (32.8–41.8)	43.1 (37.6–48.6)	31.1 (27.6–34.7)	38.7 (35.6–41.7)
Uruguay	30.4 (26.4–34.5)	45.7 (43.5–47.9)	15.6 (13.2–18.1)	34.1 (31.4–36.8)
Bahamas	62.7 (57.2–68.2)	42.2 (37.5–47)	26.2 (19.9–32.4)	38.1 (31.7–44.4)
El Salvador	39.8 (36.2–43.4)	41.5 (36.1–46.9)	21.7 (17.1–26.3)	34.1 (28.4–39.7)
Curacao	23.9 (20.5–27.4)	50.5 (47.4–53.5)	38.2 (34.2–42.3)	39.5 (36.6–42.3)
Paraguay	19 (15.7–22.2)	48.9 (45.7–52.1)	24.1 (20.9–27.3)	34.3 (30.2–38.4)
Dominican Republic	40.8 (34.8–46.8)	54.9 (47.9–61.8)	25.3 (21.7–28.9)	28.9 (24.3–33.5)
Eastern Mediterranean region				
Tajikistan	35.5 (28.6–42.3)	22.1 (17.1–27.1)	17.2 (11.9–22.6)	–
Macedonia	39.1 (29.4–48.8)	41.1 (35.6–46.5)	17.8 (13.3–22.3)	–
Djibouti	32.6 (27.8–37.5)	55 (50.6–59.4)	30.7 (25.6–35.7)	–
South-East Asian Region				
Indonesia	21.5 (16.6–26.4)	21.2 (17.5–24.9)	19.3 (16–22.5)	15.3 (12.6–18)
Thailand	28.6 (24–33.1)	37.5 (33.3–41.7)	21.2 (16.7–25.8)	17.7 (14.8–20.6)
Bangladesh	37.1 (23.6–50.5)	-	30.6 (16.6–44.5)	14.8 (7.2–22.4)
Bhutan	28.4 (24.6–32.1)	42.3 (39.3–45.4)	25.8 (22.4–29.3)	30.1 (26.7–33.4)
Timor Leste	22.5 (18.1–26.9)	30.6 (25.7–35.6)	25.8 (21.9–29.7)	19.8 (16.1–23.5)
Nepal	23 (17.9–28.1)	18.7 (13.5–23.9)	13.3 (9.5–17.2)	11.5 (8.2–14.8)
Western Pacific Region				
Cambodia	8.6 (5.3–12)	8.8 (4.8–12.7)	6.1 (4.4–7.8)	7.5 (5.5–9.4)
Fiji	29.3 (24.9–33.7)	39.9 (35–44.8)	29.8 (24.4–35.3)	29.7 (24.6–34.7)
Kiribati	34 (29.6–38.4)	39.2 (34.5–43.9)	51.5 (45–58)	31.1 (26–36.1)
Malaysia	15.8 (13.3–18.3)	16.9 (14.2–19.7)	19.3 (16.3–22.3)	10.6 (8.8–12.3)
Mongolia	19.1 (16–22.1)	35.8 (31.4–40.1)	29.4 (25.4–33.5)	31.6 (26.4–36.9)
Nauru	36.5 (36.5–36.5)	48.2 (48.2–48.2)	30.3 (30.3–30.3)	–
Samoa	23 (17.8–28.2)	32 (25.2–38.8)	26.2 (20.5–31.9)	25.2 (20.6–29.7)
Vanuatu	15.9 (12.3–19.5)	31.8 (26.7–36.9)	26.9 (21.8–32.1)	19.8 (15.7–24)
Vietnam	15.2 (10–20.4)	23.4 (16.9–29.8)	28.8 (20.7–36.9)	15.5 (9.8–21.2)
Brunei	17.4 (12.8–22)	17.9 (11.6–24.1)	18.9 (13–24.7)	16.2 (11.7–20.8)
Tuvalu	29.8 (29.8–29.8)	34.5 (34.5–34.5)	28.7 (28.7–28.7)	20.9 (20.9–20.9)
French Polynesia	29.3 (24.7–34)	47.3 (42.9–51.7)	45.8 (41.3–50.3)	38.8 (36.2–41.4)
Laos	8.3 (4.4–12.1)	28.8 (21.9–35.6)	21 (15.6–26.3)	10 (7.8–12.2)
Tokelau	43.3 (30.8–55.8)	53.7 (43.2–64.2)	60.2 (44.3–76.1)	50 (46.1–53.9)
Wallis and Futuna	36.4 (28.4–44.5)	51.2 (44.4–58)	39.1 (32.6–45.6)	37.7 (30.7–44.8)

In Africa, the highest proportion of sexual intercourse before age 14 (36.5%) and practicing sexual intercourse without condom use (32.2%) were reported. In the Americas, the highest rates of multiple sexual partners (46.7%) and sexual intercourse without birth control methods (32.7%) were reported. In the Western Pacific region, the lowest proportion of sexual intercourse before age 14 (14.9%) and practicing sexual intercourse without birth control methods (14.6%) were reported.

There were country-level differences within the regions. In Africa, Kenya reported the highest rate of sexual intercourse before age 14 at 68.9%, followed by Senegal at 65.3% and Zambia at 53.2%. Benin reported the highest rates of multiple sexual partners (61.2%), while Seychelles and Kenya reported the highest proportions of intercourse without birth control (48.7%) and without condom use (49.2%), respectively. Liberia reported the lowest rate of sexual intercourse before age 14 (15.1%), while Malawi had the lowest rates of multiple sexual partners (22.5%), and intercourse without condom use (13.8%).

In the Americas, Saint Vincent and the Grenadines reported the highest rates of sexual intercourse before age 14 (68.8%). Jamaica, Chile, and Dominica reported the highest rates of multiple sexual partners (63.6%), intercourse without condom use (39.0%), and without birth control methods (52.8%), respectively. Paraguay reported the lowest rates of sexual intercourse before age 14 (19.0%). Honduras, Uruguay, and Costa Rica reported the lowest rates of multiple sexual partners (34.7%), intercourse without condom use (15.6%), and without birth control methods (25.0%), respectively.

In the Eastern Mediterranean Region, Macedonia reported the highest rates of sexual intercourse before age 14 (39.1%). Djibouti had the highest proportions of having multiple sexual partners (55.0%) and intercourse without condom use (30.7%). Djibouti reported the lowest rates of sexual intercourse before age 14 (32.6%). Tajikistan had the lowest rates of multiple sexual partners (22.1%) and intercourse without condom use (17.2%). No data on birth control methods were available.

In the South-East Asian Region, Bangladesh reported the highest rates of sexual intercourse before age 14 (37.1%) and intercourse without condom use (30.6%). Bhutan reported the highest rates of multiple sexual partners (42.3%) and intercourse without birth control methods (30.1%). Nepal recorded the lowest rates of multiple sexual partners (18.7%), intercourse without condom use (13.3%), and no birth control methods (11.5%). Indonesia reported the lowest rates of sexual intercourse before age 14 (21.5%).

In the Western Pacific Region, Tokelau recorded the highest rates for sexual intercourse before age 14 (43.3%), multiple sexual partners (53.7%), intercourse without condom use (60.2%), and no birth control methods (50.0%). Laos recorded the lowest rates of sexual intercourse before age 14 (8.3%). Cambodia reported the lowest rates of multiple sexual partners (8.8%), intercourse without condom use (6.1%), and no birth control methods (7.5%).

The estimated prevalence of unsafe sex factors against the prevalence of ever having sex with sizes representing the corresponding number of adolescents of the countries at risk is presented in Supplement 1 (data table) and Supplement 2 Figs. 1–6. In Africa, the prevalence of ever-had sex was high in Mozambique, Benin, and Liberia. Benin had both a high prevalence of ever-had sex and unsafe sex. Senegal had a low prevalence of ever had sex although had high unsafe sex. In the Americas, the prevalence of ever-had sex was high in Jamaica, Dominica, and Grenada. Jamaica and Dominica had both high ever had sex and unsafe sex. Peru had low ever-had sex but high unsafe sex. In the Eastern Mediterranean, the prevalence of ever-had sex was high in Djibouti. Djibouti had both high ever had sex and unsafe sex. In Southeast Asia, the prevalence of ever-had sex was high in Timor Leste, Nepal, and Thailand. Timor Leste and Thailand both reported high ever-had sex and unsafe sex. Bangladesh had both low ever had sex but high unsafe sex. In the Western Pacific, the prevalence of ever-had sex was high in French Polynesia, Nauru, and Tokelau. Tokelau had both high ever had sex and unsafe sex. Although the prevalence was relatively low, Indonesia has the highest total number of adolescents who had unsafe sex due to its large population size (multiple sex partners = 21.2%, 9.7 million cases, no condom use = 19.3%, 8.8 million cases, sex before age 14 = 21.5%, 9.8 million cases, no birth control = 15.3%, 7.0 million cases).

## Discussion

We analyzed data from 68 countries using the GSHS and found a significant prevalence of unsafe sexual practices. The rates for unsafe sexual practice (multiple sexual partners, early sexual intercourse, having sex without a condom, and no birth control during intercourse) varied across regions. Our results are notably lower than those from a study involving 43 low- and middle-income countries (e.g., 52.0% with multiple partners, 44.2% without condom use) (Smith et al., 2022) and four Caribbean countries study (Pengpid & Peltzer, 2020) using the same GSHS data. The variation in prevalence is primarily due to differences in participant age ranges, year of the survey, and number of included countries. The previous study in low- and middle-income countries focused on ages 12–15, while the Caribbean countries survey only covered the years 2016 and 2017. Unlike the previous study, our research covers a broader scope with more countries, varied survey years, and a wider age range of participants.

The early initiation of sexual practice was common in the Americas and Africa. However, the prevalence of unsafe sex was notably higher in Africa, which may be attributed to a combination of cultural influences and risky school environments (Ngidi & Essack, 2022). Such behavior could affect students’ physical and psychological health (Mahumud et al., 2022; Odimegwu et al., 2019; Seff et al., 2021), underscoring the importance of developing targeted interventions aimed at improving the wellbeing of adolescents in these regions and the need to commit to the SDGs, including universal health coverage (SDG Target 3.8) (World Health Organization, 2022c).

The African Region also had a higher proportion of sexual practices without condom use. The sub-Saharan African countries are home to more than two-thirds of people in the world living with HIV. Condom use is known to be one of the most critical components of HIV prevention. The majority religion in Sub-Saharan African countries is Christianity, in which there is a moral objection to condom use and other contraceptive use in some south African countries (Muula, 2010). This cultural influence could be one of the major reasons why the African Region had the highest prevalence of sex without a condom. It has also been shown that in these countries, misinformation about condom efficacy is high (Winskell et al., 2011). The UN considers sexual education as a human right. The strategies and efforts put in place should include additional efforts on sexual and reproductive education in school settings too.

There is a substantial heterogeneity in the prevalence of sexual practice without birth control, with the highest prevalence recorded in the American region. The high prevalence in the American region could be explained by socioeconomic inequality where there are large wealth inequalities in the contraceptive use (Leon et al., 2019). Proper sex education is essential to lower the prevalence of unsafe sex in the American region as sex is seen as a taboo topic that is not often discussed. This study supports the call for action to meet the sustainable development goals SDGs, target 3.7 to “ensure universal access to sexual and reproductive health-care services, including integration of reproductive health into national strategies and programmes” by 2030 (United Nations, 2019).

This study indicates the need for future action to reduce unsafe sex among students. There are more than 21 million pregnancies each year among adolescents in low- and middle-income countries and 50% were unintended (Sully et al., 2020; World Health Organization, 2022b). Therefore, school-based (Maina et al., 2021) and family-based interventions (Downing et al., 2011) are recommended to help reduce the prevalence of unsafe sex among students and to meet the UN 2030 SDG Goals (World Health Organization, 2021). Acknowledging that progress has been made to reduce unsafe sexual practices among students, the UN SDG Goals (Cluver et al., 2019), WHO’s Global Accelerated Action for the Health of Adolescents (AA-HA!) (World Health Organization, 2017), and the UN Universal Declaration of Human Rights continues to call upon further action to improve sexual health in adolescents around the world (World Health Organization, 2022a).

The GSHS is cross-sectional, and therefore, the results cannot infer causation. The data were collected on self-reports, possibly leading to under or over-reporting of adolescents’ sexual behavior. Some surveys included in this study date back to 2003 indicating that some data would not be representative of adolescents today. There were limited countries included (*n* = 3) for the Mediterranean region as the data were unavailable for the remaining countries in this region. Future studies could explore country-level correlates of unsafe sex. As this study was conducted at the school level, the data were not representative of adolescent school dropouts which have been associated with greater levels of sexual risk and vulnerability, especially in girls (Anderson & Pörtner, 2014).

In conclusion, our findings revealed that the prevalence of unsafe sex behaviors was highest in the American and African regions, and some countries in Southeast Asia had a relatively low prevalence but a large number of total cases due to large population sizes. This study underscores the necessity for targeted interventions and policy measures tailored to specific regions to address and reduce the prevalence of unsafe sexual behaviors in those regions. Global efforts to prevent unsafe sex and attributable disease burden in adolescents also need to consider both prevalence rates and cases to meet the UN 2030 SDG Goals.

### Supplementary Information

Below is the link to the electronic supplementary material.

Supplementary file1 (DOCX 32 KB)

Supplementary file2 (DOCX 1870 KB)

## Data Availability

The authors confirm that the data supporting the findings of this study are available within the article and its supplementary materials.
