# Supplementary material for: Regional and Country Prevalence Estimates of Unsafe Sex Among Adolescents in 68 Low-Income and Middle-Income Countries
Source: Arch Sex Behav. 2024 Apr 18;53(6):2337–46. doi: 10.1007/s10508-024-02861-1 (PMC11176239; doi:10.1007/s10508-024-02861-1)
Supplement: Supplementary file 1 — Supplementary file1 (DOCX 32 KB) [file 10508_2024_2861_MOESM1_ESM.docx]

# Supplement 1

## Table S1. Self-reports of sexual intercourse by unsafe sex behaviours and estimated population size of adolescents in each country

|  | Country | Population size in country aged 10-19 | Proportion reported ever had sex | Among adolescents who self-reported that they ever had sex | | | | | | | | |
| --- | --- | --- | --- | --- | --- | --- | --- | --- | --- | --- | --- | --- |
|  |  |  |  | Multiple sex partners | | No condom use | | Sex before age 14 | | No birth control | |  |
|  |  |  |  | % | Estimated n in population | % | Estimated n in population | % | Estimated n in population | % | Estimated n in population |  |
| Africa | |  |  |  |  |  |  |  |  |  |  |  |
|  | Benin | 2,638,174 | 49.9 | 61.2 | 1,614,562 | 47.3 | 1,246,975 | 23.4 | 618,491 | 41.2 | 1,087,199 |  |
|  | Botswana | 405,093 | 19.2 | 45.6 | 184,722 | 27.5 | 111,307 | 43.7 | 177,166 | -- | -- |  |
|  | Ghana | 5,373,444 | 31.7 | 33.8 | 1,816,224 | 33.2 | 1,784,854 | 25.6 | 1,377,724 | 27.8 | 1,495,139 |  |
|  | Kenya | 8,126,909 | 27.0 | -- | -- | 49.2 | 4,001,397 | 68.9 | 5,599,985 | -- | -- |  |
|  | Malawi | 3,531,691 | 22.9 | 22.5 | 794,630 | 13.8 | 487,313 | 40.2 | 1,419,351 | -- | -- |  |
|  | Mauritania | 731,596 | 29.7 | 33.9 | 248,011 | 21.5 | 157,006 | 33.8 | 246,956 | 18.5 | 135,132 |  |
|  | Mauritius | 173,270 | 19.7 | 38.9 | 67,402 | 35.2 | 61,075 | 27.0 | 46,708 | 39.0 | 67,528 |  |
|  | Namibia | 537,050 | 47.1 | 49.3 | 264,766 | 19.5 | 104,756 | 32.9 | 176,622 | 30.7 | 164,985 |  |
|  | Senegal | 2,625,229 | 22.0 | 58.2 | 1,527,883 | 37.5 | 985,217 | 65.3 | 1,715,464 |  |  |  |
|  | Seychelles | 12,290 | 37.9 | 53.7 | 6,600 | 38.8 | 4,770 | 51.4 | 6,319 | 48.7 | 5,983 |  |
|  | Swaziland | 218,003 | 26.0 | 44.8 | 97,665 | 25.0 | 54,596 | -- | -- | 36.9 | 80,460 |  |
|  | Uganda | 6,625,584 | 20.4 | 48.1 | 3,186,906 | 32.4 | 2,146,100 | 46.3 | 3,066,175 | -- | -- |  |
|  | Tanzania | 11,914,347 | 19.0 | 26.9 | 3,204,959 | 29.3 | 3,491,047 | 37.2 | 4,427,836 | 17.6 | 2,096,973 |  |
|  | Zambia | 2,725,574 | 22.4 | 47.4 | 1,291,922 | 34.6 | 942,386 | 53.2 | 1,450,084 | -- | -- |  |
|  | Liberia | 1,188,315 | 49.8 | 45.1 | 535,930 | 27.5 | 327,367 | 15.1 | 179,929 | 20.5 | 243,531 |  |
|  | Mozambique | 6,141,938 | 50.7 | 40.8 | 2,505,911 | 17.5 | 1,075,361 | 28.4 | 1,747,013 | 23.4 | 1,436,612 |  |
| Americas | |  |  |  |  |  |  |  |  |  |  |  |
|  | Anguilla | 2,470 | 32.2 | 47.8 | 1,181 | 28.4 | 702 | 44.4 | 1,097 | 39.4 | 972 |  |
|  | Antigua and Barbuda | 15,640 | 33.9 | 62.6 | 9,791 | 27.7 | 4,333 | 67.3 | 10,527 | 51.3 | 8,031 |  |
|  | Argentina | 6,801,385 | 40.0 | 48.5 | 3,298,672 | 19.6 | 1,333,663 | 41.6 | 2,830,736 | 30.8 | 2,097,806 |  |
|  | Barbados | 39,206 | 33.3 | 48.3 | 18,936 | 29.0 | 11,376 | 51.3 | 20,095 | 45.8 | 17,941 |  |
|  | Belize | 68,717 | 23.5 | 52.7 | 36,214 | 24.3 | 16,684 | 45.3 | 31,148 | 37.1 | 25,483 |  |
|  | Bolivia | 2,139,220 | 24.9 | 39.4 | 842,853 | 29.4 | 629,219 | 30.5 | 651,852 | 29.2 | 624,308 |  |
|  | British Virgin Islands | 4,174 | 37.8 | 58.4 | 2,438 | 23.9 | 996 | 58.0 | 2,422 | 35.2 | 1,470 |  |
|  | Cayman Islands | 6,652 | 37.0 | 52.8 | 3,512 | 24.0 | 1,599 | 64.7 | 4,303 | -- | -- |  |
|  | Chile | 2,517,122 | 37.9 | 44.5 | 1,120,119 | 39.0 | 981,612 | 23.4 | 588,143 | 33.5 | 842,199 |  |
|  | Costa Rica | 819,881 | 23.5 | 44.7 | 366,487 | 32.0 | 262,550 | 36.8 | 301,757 | 25.0 | 204,602 |  |
|  | Dominica | 12,357 | 45.0 | 59.0 | 7,291 | 29.7 | 3,665 | 60.9 | 7,527 | 52.8 | 6,529 |  |
|  | Grenada | 19,623 | 42.6 | 55.2 | 10,832 | 30.4 | 5,960 | 61.7 | 12,104 | -- | -- |  |
|  | Guatemala | 3,453,902 | 19.5 | 41.9 | 1,447,185 | 29.6 | 1,021,288 | 31.5 | 1,089,302 | 31.8 | 1,099,947 |  |
|  | Guyana | 167,484 | 31.8 | 46.8 | 78,383 | 23.4 | 39,263 | 47.6 | 79,698 | -- | -- |  |
|  | Honduras | 1,837,973 | 24.7 | 34.7 | 637,777 | 23.5 | 431,021 | 39.5 | 725,819 | 26.8 | 492,103 |  |
|  | Jamaica | 503,421 | 46.8 | 63.6 | 320,176 | 30.4 | 152,997 | 53.7 | 270,325 | 44.0 | 221,262 |  |
|  | Peru | 5,613,658 | 19.4 | 46.9 | 2,632,806 | 32.6 | 1,828,105 | 41.8 | 2,348,491 | 41.3 | 2,320,366 |  |
|  | Saint Kitts and Nevis | 8,373 | 36.1 | 58.5 | 4,898 | 37.1 | 3,107 | 52.5 | 4,399 | 46.4 | 3,882 |  |
|  | Saint Lucia | 29,162 | 34.2 | 55.5 | 16,185 | 37.4 | 10,895 | 60.0 | 17,487 | -- | -- |  |
|  | Saint Vincent and the Grenadines | 19,123 | 39.9 | 55.2 | 10,556 | 30.8 | 5,889 | 68.8 | 13,153 | -- | -- |  |
|  | Suriname | 104,874 | 32.3 | 50.3 | 52,752 | 30.0 | 31,437 | 33.1 | 34,737 | 40.4 | 42,334 |  |
|  | Trinidad and Tobago | 188,698 | 26.2 | 43.1 | 81,329 | 31.1 | 58,775 | 37.3 | 70,365 | 38.7 | 72,981 |  |
|  | Uruguay | 535,403 | 33.7 | 45.7 | 244,679 | 15.6 | 83,711 | 30.4 | 162,885 | 34.1 | 182,623 |  |
|  | Bahamas | 53,315 | 28.2 | 42.2 | 22,499 | 26.2 | 13,948 | 62.7 | 33,434 | 38.1 | 20,292 |  |
|  | El Salvador | 1,269,700 | 22.4 | 41.5 | 526,926 | 21.7 | 275,806 | 39.8 | 505,428 | 34.1 | 432,714 |  |
|  | Curacao | 20,584 | 36.9 | 50.5 | 10,395 | 38.2 | 7,867 | 23.9 | 4,923 | 39.5 | 8,124 |  |
|  | Paraguay | 1,284,147 | 28.6 | 48.9 | 627,948 | 24.1 | 309,420 | 19.0 | 243,476 | 34.3 | 440,819 |  |
|  | Dominican Republic | 1,949,312 | 40.7 | 54.9 | 1,070,172 | 25.3 | 493,657 | 40.8 | 794,803 | 28.9 | 563,425 |  |
| Eastern Mediterranean | |  |  |  |  |  |  |  |  |  |  |  |
|  | Tajikistan | 1,670,147 | 8.7 | 22.1 | 369,102 | 17.2 | 287,529 | 35.5 | 592,495 | -- | -- |  |
|  | North Macedonia | 296,272 | 15.9 | 41.1 | 121,768 | 17.8 | 52,649 | 39.1 | 115,903 | -- | -- |  |
|  | Djibouti | 171,676 | 34.7 | 55.0 | 94,422 | 30.7 | 52,668 | 32.6 | 56,026 | -- | -- |  |
| Southeast Asia | |  |  |  |  |  |  |  |  |  |  |  |
|  | Indonesia | 45,795,922 | 5.8 | 21.2 | 9,708,735 | 19.3 | 8,827,668 | 21.5 | 9,842,231 | 15.3 | 7,021,019 |  |
|  | Thailand | 8,869,429 | 19.1 | 37.5 | 3,326,036 | 21.2 | 1,881,685 | 28.6 | 2,533,322 | 17.7 | 1,571,956 |  |
|  | Bangladesh | 31,492,746 | 8.2 | -- | -- | 30.6 | 9,630,198 | 37.1 | 11,668,440 | 14.8 | 4,650,880 |  |
|  | Bhutan | 154,141 | 17.1 | 42.3 | 65,202 | 25.8 | 39,815 | 28.4 | 43,718 | 30.1 | 46,323 |  |
|  | Timor Leste | 291,081 | 22.1 | 30.6 | 89,071 | 25.8 | 75,021 | 22.5 | 65,510 | 19.8 | 57,625 |  |
|  | Nepal | 6,422,331 | 19.2 | 18.7 | 1,200,976 | 13.3 | 857,054 | 23.0 | 1,476,603 | 11.5 | 738,266 |  |
| Western pacific | |  |  |  |  |  |  |  |  |  |  |  |
|  | Cambodia | 2,755,283 | 11.6 | 8.8 | 242,465 | 6.1 | 168,003 | 8.6 | 238,004 | 7.5 | 206,473 |  |
|  | Fiji | 152,196 | 21.3 | 39.9 | 60,726 | 29.8 | 45,400 | 29.3 | 44,547 | 29.7 | 45,188 |  |
|  | Kiribati | 23,036 | 23.5 | 39.2 | 9,030 | 51.5 | 11,866 | 34.0 | 7,826 | 31.1 | 7,153 |  |
|  | Malaysia | 5,697,857 | 8.2 | 16.9 | 962,938 | 19.3 | 1,101,362 | 15.8 | 898,979 | 10.6 | 601,124 |  |
|  | Mongolia | 477,943 | 15.1 | 35.8 | 171,104 | 29.4 | 140,661 | 19.1 | 91,118 | 31.6 | 151,258 |  |
|  | Nauru | 1,683 | 31.7 | 48.2 | 811 | 30.3 | 510 | 36.5 | 614 | -- | -- |  |
|  | Samoa | 41,805 | 25.0 | 32.0 | 13,378 | 26.2 | 10,937 | 23.0 | 9,623 | 25.2 | 10,518 |  |
|  | Vanuatu | 64,467 | 26.8 | 31.8 | 20,501 | 26.9 | 17,363 | 15.9 | 10,267 | 19.8 | 12,774 |  |
|  | Vietnam | 15,475,948 | 6.7 | 23.4 | 3,621,372 | 28.8 | 4,461,607 | 15.2 | 2,351,771 | 15.5 | 2,394,299 |  |
|  | Brunei | 69,353 | 11.4 | 17.9 | 12,414 | 18.9 | 13,078 | 17.4 | 12,101 | 16.2 | 11,251 |  |
|  | Tuvalu | 2,069 | 18.6 | 34.5 | 714 | 28.7 | 593 | 29.8 | 617 | 20.9 | 432 |  |
|  | French Polynesia | 44,817 | 40.1 | 47.3 | 21,198 | 45.8 | 20,531 | 29.3 | 13,139 | 38.8 | 17,382 |  |
|  | Laos | 1,556,808 | 14.5 | 28.8 | 448,361 | 21.0 | 326,701 | 8.3 | 128,532 | 10.0 | 155,226 |  |
|  | Tokelau | 327 | 30.2 | 53.7 | 176 | 60.2 | 197 | 43.3 | 142 | 50.0 | 163 |  |
|  | Wallis and Futuna | 2,652 | 26.8 | 51.2 | 1,358 | 39.1 | 1,037 | 36.4 | 966 | 37.7 | 1,001 |  |
