# Supplementary material for: Regional and Country Prevalence Estimates of Unsafe Sex Among Adolescents in 68 Low-Income and Middle-Income Countries
Source: Arch Sex Behav. 2024 Apr 18;53(6):2337–46. doi: 10.1007/s10508-024-02861-1 (PMC11176239; doi:10.1007/s10508-024-02861-1)
Supplement: Supplementary file 2 — Supplementary file2 (DOCX 1870 KB) [file 10508_2024_2861_MOESM2_ESM.docx]

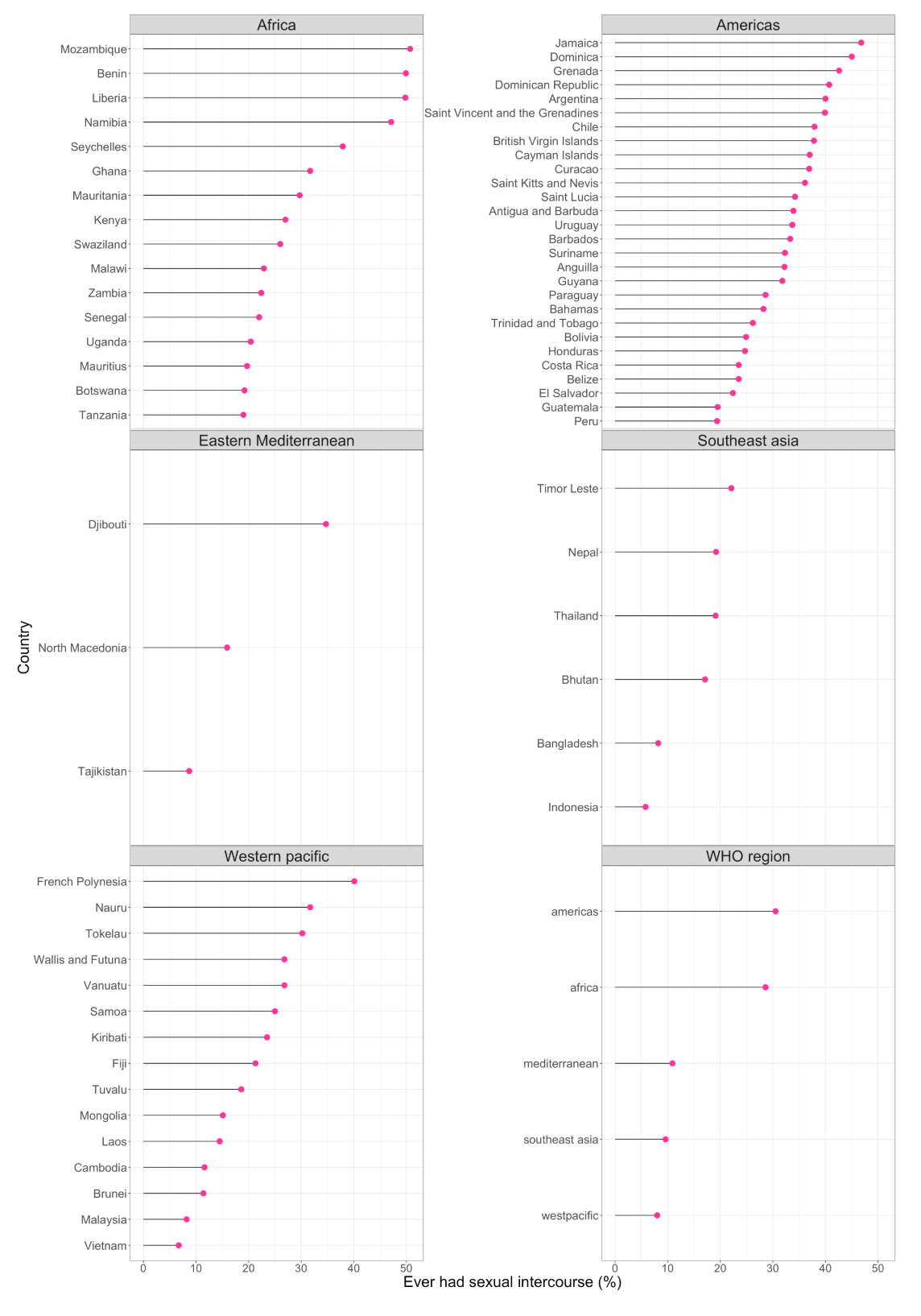


***Fig 1. Prevalence of sexual intercourse among adolescents***


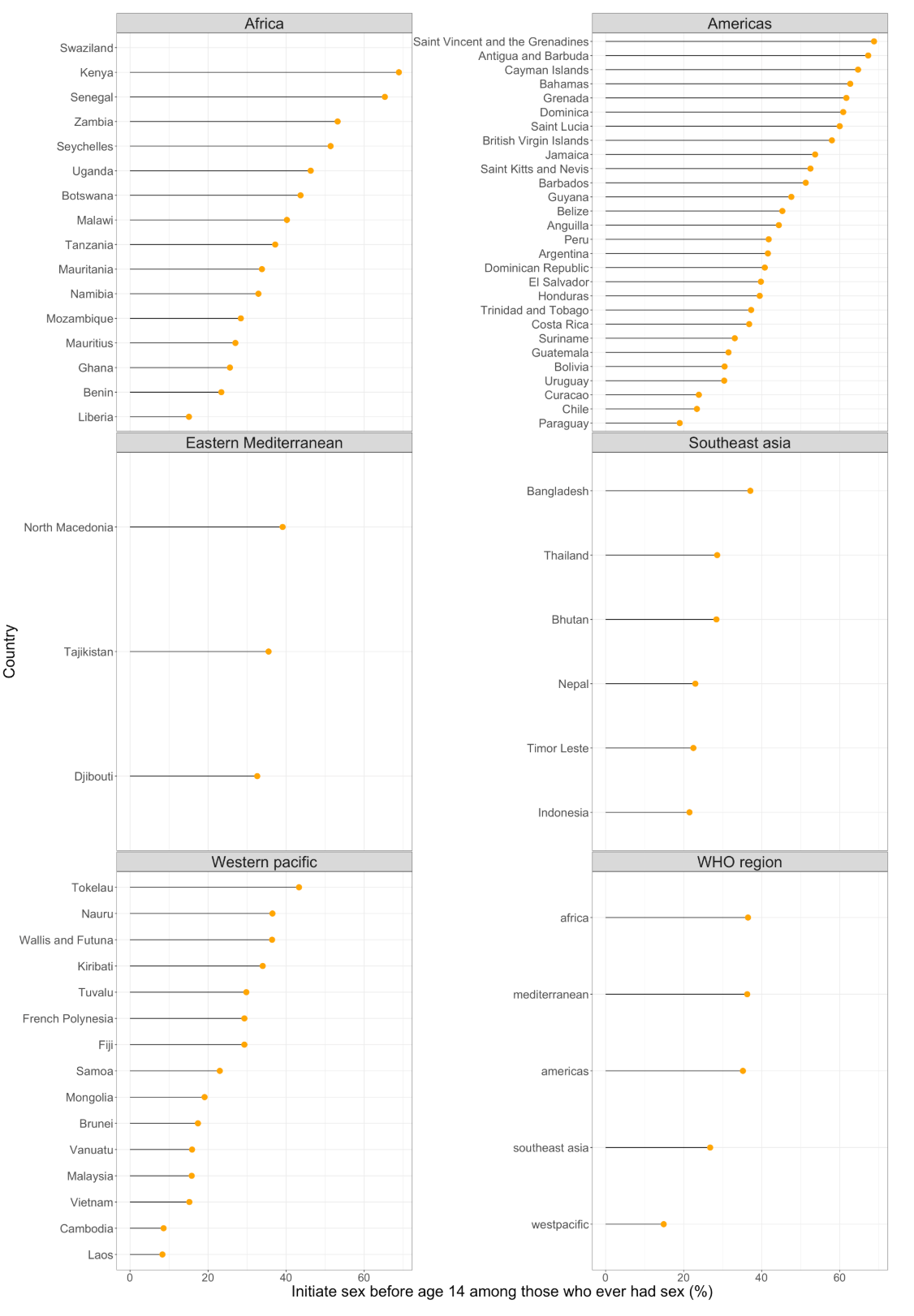


***Fig 2. Prevalence of sexual intercourse initiation before age 14 among adolescents***


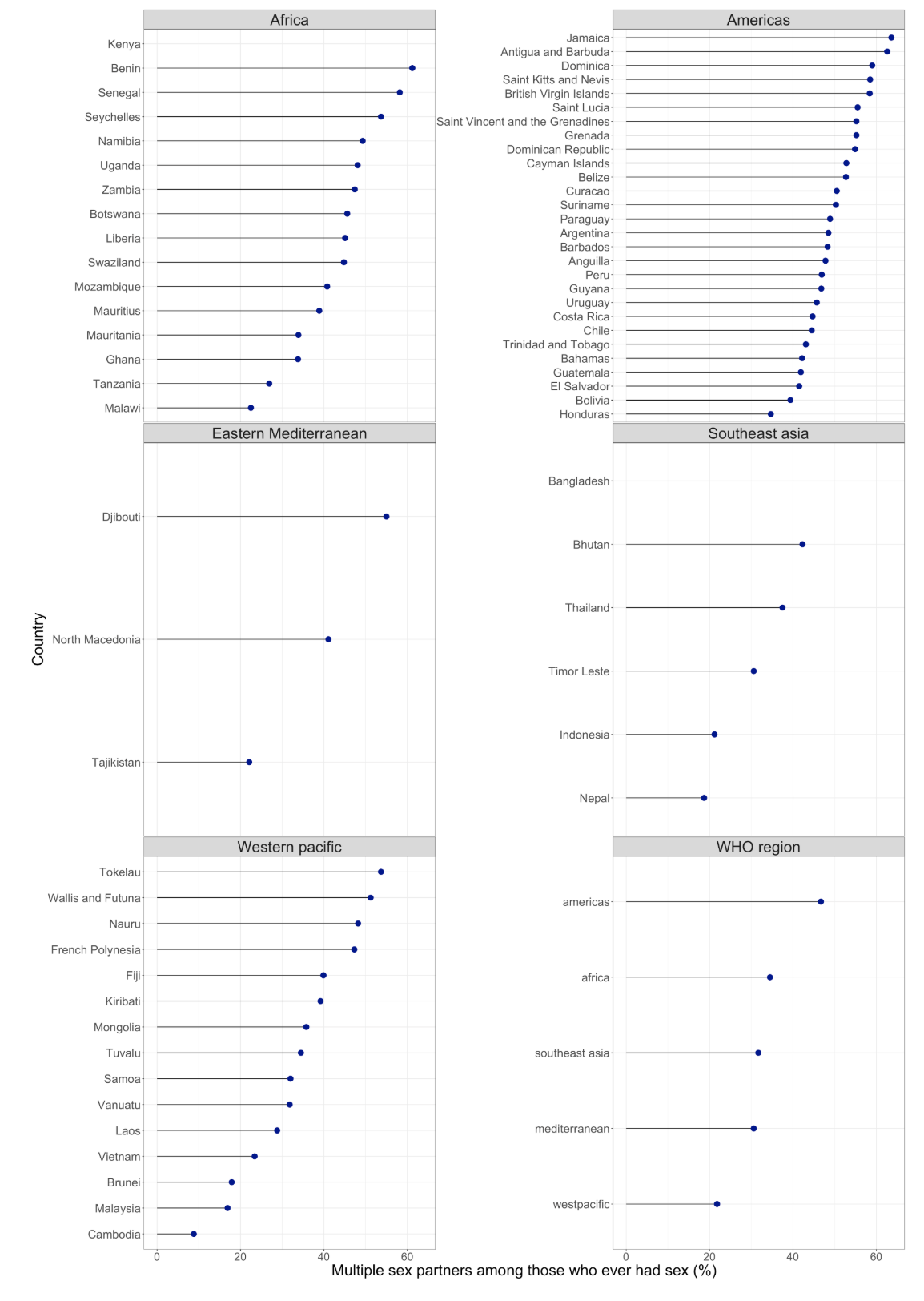


***Fig 3. Prevalence of multiple sex partners among adolescents***


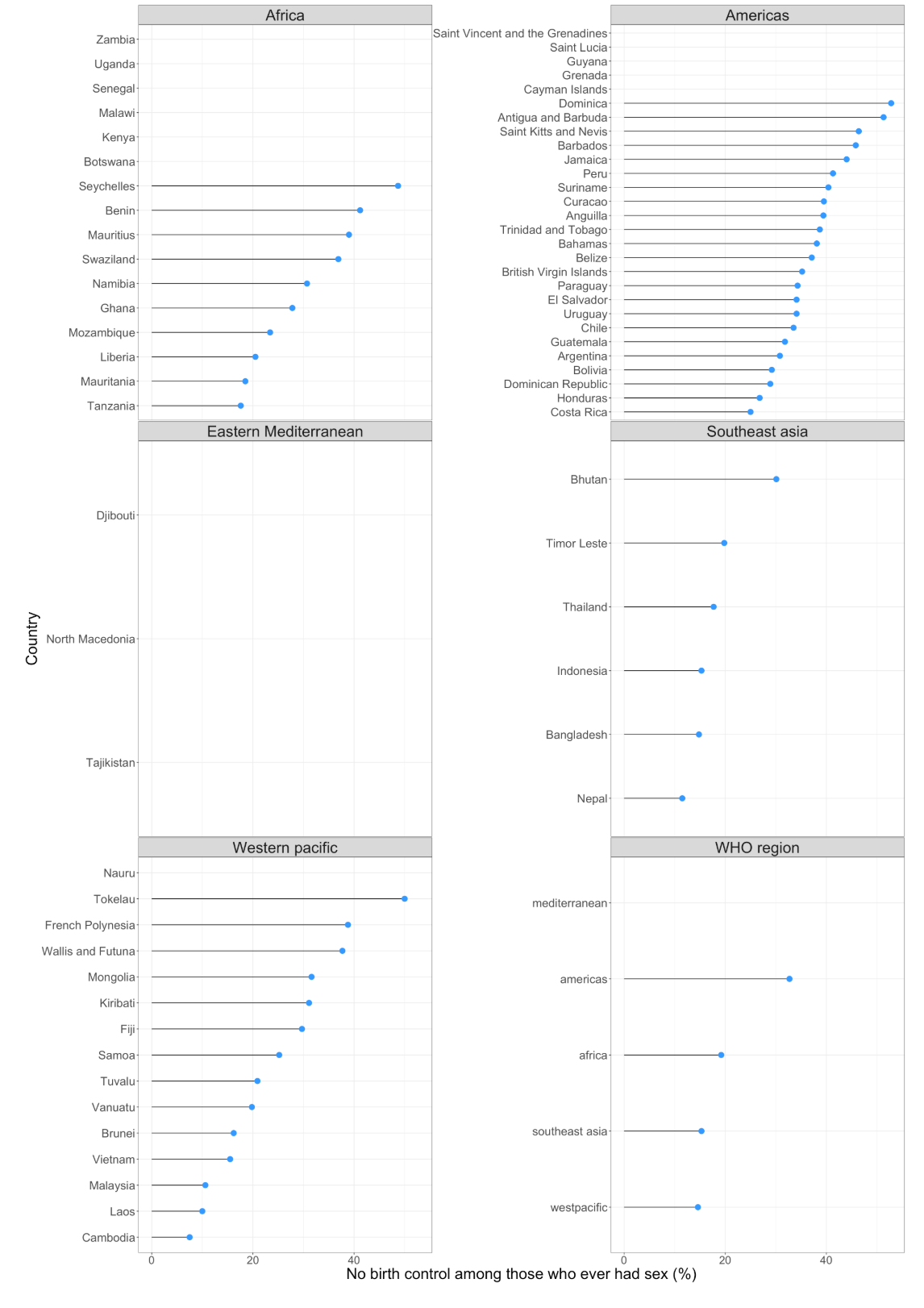


***Fig 4. Prevalence of non-use birth control methods during sexual intercourse among adolescents***


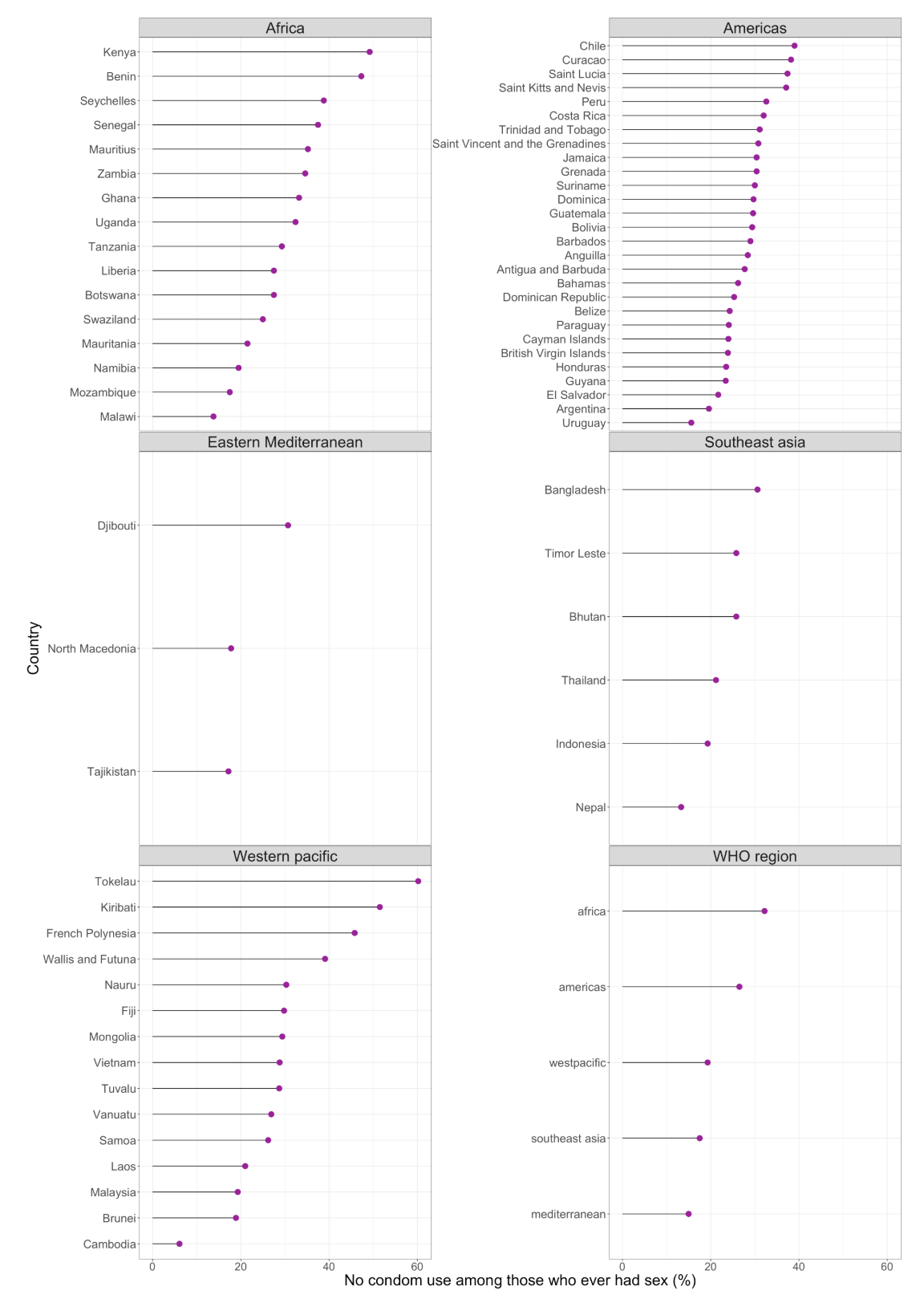


***Fig 5. Prevalence of non-use of condom during sexual intercourse among adolescents***


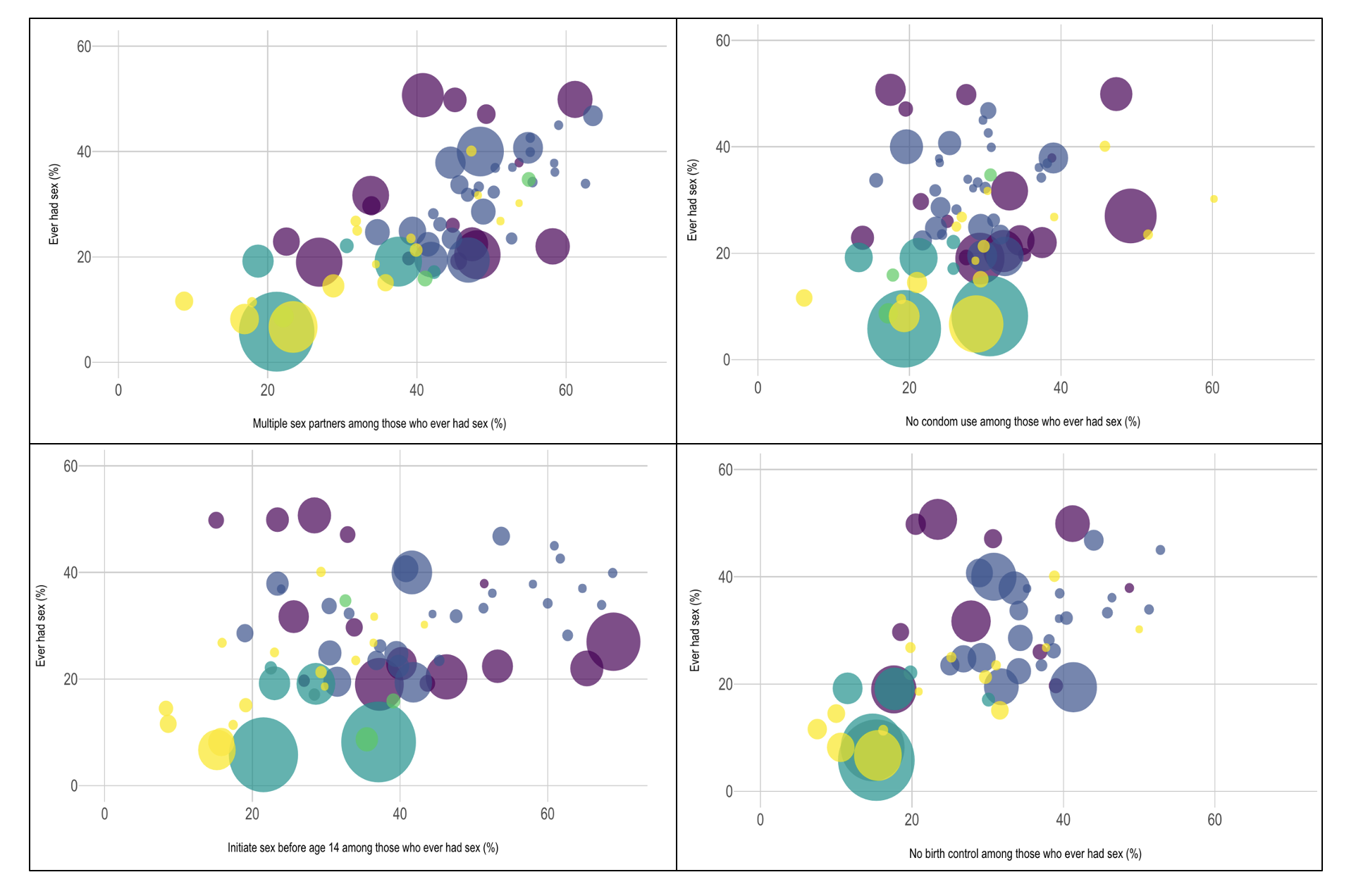


***Figure 6. Bubble plots of ever had sexual intercourse (y-axis) and sexual risk behaviour (x-axis) with bubble size proportional to population (10-19yrs) with sexual risk behaviour***
